# Family physician leadership during the COVID-19 pandemic: roles, functions and key supports

**DOI:** 10.1108/LHS-03-2022-0030

**Published:** 2022-07-27

**Authors:** Maria Mathews, Dana Ryan, Lindsay Hedden, Julia Lukewich, Emily Gard Marshall, Judith Belle Brown, Paul S. Gill, Madeleine McKay, Eric Wong, Stephen J. Wetmore, Richard Buote, Leslie Meredith, Lauren Moritz, Sarah Spencer, Maria Alexiadis, Thomas R. Freeman, Aimee Letto, Bridget L. Ryan, Shannon L. Sibbald, Amanda Lee Terry

**Affiliations:** Department of Family Medicine, Schulich School of Medicine and Dentistry, Western University, London, Canada; Department of Family Medicine, Schulich School of Medicine and Dentistry, Western University, London, Canada; Faculty of Health Sciences, Simon Fraser University, Burnaby, Canada; Faculty of Nursing, Memorial University, St. John's, Canada; Department of Family Medicine Primary Care Research Unit, Dalhousie University, Halifax, Canada; Department of Family Medicine, Schulich School of Medicine and Dentistry, Western University, London, Canada; Department of Family Medicine, Schulich School of Medicine and Dentistry, Western University, London, Canada; Doctors Nova Scotia, Dartmouth, Canada; Department of Family Medicine, Schulich School of Medicine and Dentistry, Western University, London, Canada; Department of Family Medicine, Schulich School of Medicine and Dentistry, Western University, London, Canada; Department of Family Medicine Primary Care Research Unit, Dalhousie University, Halifax, Canada; Department of Family Medicine, Schulich School of Medicine and Dentistry, Western University, London, Canada; Department of Family Medicine Primary Care Research Unit, Dalhousie University, Halifax, Canada; Faculty of Health Sciences, Simon Fraser University, Burnaby, Canada; Department of Family Practice, Nova Scotia Health Authority, Halifax, Canada; Department of Family Medicine, Schulich School of Medicine and Dentistry, Western University, London, Canada; Newfoundland and Labrador Medical Association, St. John's, Canada; Department of Family Medicine, Schulich School of Medicine and Dentistry, Western University, London, Canada; Department of Family Medicine, Schulich School of Medicine and Dentistry, Western University, London, Canada; Department of Family Medicine, Schulich School of Medicine and Dentistry, Western University London, Canada, and Department of Epidemiology and Biostatistics, Schulich School of Western University Medicine and Dentistry, Western University, London, Canada

**Keywords:** Leadership, Primary care, Family physician, Pandemic response, COVID-19, Policy planning, Qualitative research

## Abstract

**Purpose:**

Strong leadership in primary care is necessary to coordinate an effective pandemic response; however, descriptions of leadership roles for family physicians are absent from previous pandemic plans. This study aims to describe the leadership roles and functions family physicians played during the COVID-19 pandemic in Canada and identify supports and barriers to formalizing these roles in future pandemic plans.

**Design/methodology/approach:**

This study conducted semi-structured qualitative interviews with family physicians across four regions in Canada as part of a multiple case study. During the interviews, participants were asked about their roles during each pandemic stage and the facilitators and barriers they experienced. Interviews were transcribed and a thematic analysis approach was used to identify recurring themes.

**Findings:**

Sixty-eight family physicians completed interviews. Three key functions of family physician leadership during the pandemic were identified: conveying knowledge, developing and adapting protocols for primary care practices and advocacy. Each function involved curating and synthesizing information, tailoring communications based on individual needs and building upon established relationships.

**Practical implications:**

Findings demonstrate the need for future pandemic plans to incorporate formal family physician leadership appointments, as well as supports such as training, communication aides and compensation to allow family physicians to enact these key roles.

**Originality/value:**

The COVID-19 pandemic presents a unique opportunity to examine the leadership roles of family physicians, which have been largely overlooked in past pandemic plans. This study’s findings highlight the importance of these roles toward delivering an effective and coordinated pandemic response with uninterrupted and safe access to primary care.

## Introduction

The COVID-19 pandemic demanded an unprecedented response from family physicians requiring a coordinated approach to the adaptation and adoption of activities to respond to public health directives, fulfill pandemic-related roles and continue the delivery of routine primary care services (Government of Canada, 2018; Health Canada, 2003; Ministry of Health and Long-Term Care, 2013; Ontario Ministry of Health, 2019). The ability of family physicians to rapidly adapt to the evolving conditions of a pandemic requires strong leadership; however, no provincial pandemic plans (Government of Canada, 2018) described the leadership roles needed to support an effective and coordinated pandemic response by family physicians.

Leadership is generally defined as the capacity to direct, guide or influence (Merriam-Webster, 2022). For family physicians, the CanMEDS-Family Medicine (CanMEDS-FM) competency framework notes leadership as:

[…] integral participants in health care organizations, [family physicians] actively contribute to implementing and maintaining a high-quality health care system, and take responsibility for delivering excellent patient care through their activities as clinicians, administrators, scholars, and/or teachers (Tepper and Hawrylyshyn, 2017, p. 11).

While studies have identified the benefits of involving physicians in leadership roles in the health care system (Crocitto *et al.*, 2021; Denis *et al.*, 2013; Grimes *et al.*, 2012; Jolemore and Soroka, 2017; Stoller, 2009) there is relatively little information available on family physicians’ leadership roles during a public health crisis. Existing studies of family physician leadership focus on leadership in interdisciplinary teams (Brown *et al.*, 2015; Brown *et al.*, 2021; Brown and Ryan, 2018; Szafran *et al.*, 2018), the need for more leadership training (Gallagher *et al.*, 2017; Kelly *et al.*, 2019) or physicians in academic leadership roles (Krueger *et al.*, 2017; Oandasan *et al.*, 2013; White *et al.*, 2016).

The goal of this study is to describe the leadership roles and functions of family physicians during the COVID-19 pandemic in Canada and to identify the supports and barriers that help or hinder family physicians in enacting these leadership roles. The COVID-19 pandemic presents a unique opportunity to examine the leadership roles of family physicians in the broader health care system and strengthen the development of future pandemic plans.

## Methods

We examined four regions in Canada as part of a multiple case study (Yin, 2014): the Vancouver Coastal Health region in British Columbia, the Eastern Health region of Newfoundland and Labrador, the province of Nova Scotia and the Ontario Health West region. These regions and our rationale for selecting them are described in our previously published protocol (Mathews *et al.*, 2021). In each region, we conducted semi-structured qualitative interviews from October 2020 to June 2021 with family physicians who were recruited using maximum variation sampling (Creswell, 2014) along a wide range of characteristics, including those with and without an academic appointment, different genders, primary care funding and practice models (e.g. fee-for-service, alternative payment plans, etc.), with and without team or hospital affiliations and from urban and rural communities. Fee-for-service refers to payment by service delivered while alternate payment plans include all other forms of physician payment including capitation (fee-per-patient), salary, sessional fee, etc. In each region, recruitment continued until we had sufficient data to allow for rigorous analysis and interpretation of the data (i.e. saturation) (Berg, 1995; Creswell, 2014) which was determined through team consultation.

To be included in the study, family physicians must have held a license to practice in 2020 and been either clinically active or eligible to be clinically active in their region. We included family physicians who worked in different practice settings, including long-term care facilities and hospitals. We excluded post-graduate medical residents, physicians on temporary pandemic-related practice licenses and physicians in solely academic, research or administrative roles. To recruit family physicians, research assistants in each region emailed study invitations to physicians identified from faculty lists, lists of teams and practices (e.g. family health teams, community health centers), privileging lists and provincial College of Physicians and Surgeons’ physician search portals. We also included recruitment notices in professional organisations’ newsletters, social media posts and used snowball sampling where permitted.

In each interview, we asked family physicians to describe the various pandemic-related roles they performed over different stages of the pandemic (e.g. pre-closure, closure, reopening, vaccination) and the facilitators and barriers they experienced in performing these roles, as well as other potential roles that family physicians could have filled (Appendix). We also asked questions about their background and practice characteristics (e.g. gender, years of practice, work settings, clinic roles, community size, demographics of their practice populations). Interview questions were tailored to each province to account for differences in physician roles and broader health system contexts within these different regions. Interviews were conducted via Zoom (Zoom Video Communications Inc.) or telephone, depending on participant preference. Interviews were audio-recorded and transcribed verbatim.

We analyzed transcripts and field notes taken by the interviewer that documented observations and identified themes using thematic analysis. From each region, at least two members of the research team independently read two to three transcripts to identify key words and codes, which were organized into a preliminary coding scheme. Additional transcripts were read and codes were incorporated into the developing coding scheme. Each regional team coded a set of four transcripts (one from each region), using their own coding template. We then met to compare coding, refined the meaning of each code and developed a unified template with consistent code labels and descriptions. The unified coding template was then used across regions to code all transcripts and field notes using NVivo 12 (QSR International) software designed to assist in the organization and management of qualitative data. We resolved any disagreement in coding through consensus. We used descriptive statistics to summarize participant demographic and practice characteristic data.

We took several steps to ensure the rigor of our analyses (Berg, 1995; Creswell, 2014; Guest *et al.*, 2012). We pre-tested interview questions, documented procedures, used experienced interviewers and verified meaning with the participants during interviews. We looked for negative cases and provided thick description and illustrative quotes. Furthermore, our interdisciplinary team included family physicians and public health experts, allowing us to draw on prior expert knowledge in the development of our interview guide and the interpretation of our results (Yin, 2014).

We obtained approval from the research ethics boards at Simon Fraser University and the University of British Columbia (through the harmonized research ethics platform provided by Research Ethics British Columbia), the Health Research Ethics Board of Newfoundland and Labrador, Nova Scotia Health and Western University. Participants provided informed consent before interviews were scheduled. We reduced the risk of a privacy breach and maintained participant confidentiality through secure storage of recordings, password protection of electronic files, concealment of identifying information during the transcription process and use of study number codes to identify participants.

## Results

Across the four regions, 85 family physicians expressed initial interest in participating in the study and 68 completed an interview lasting between 17 and 97 minutes (average 58 minutes). Seventeen of the physicians who initially expressed interest in the study did not complete an interview because of scheduling conflicts, ineligibility or a lack of response to follow-up. Overall, the majority of participants were women (*n* = 41; 60.3%), paid by alternate payment plans (e.g. global funding, capitation, etc.), (*n* = 46; 67.6%), had hospital privileges (*n* = 49; 73.5%) and had their main practice setting in urban communities (*n* = 44; 64.7%) (Table 1). In this article, we report findings from the codes related to leadership. Key themes describe the context in which family physicians took on pandemic-related leadership roles, the nature of leadership functions and the enablers and barriers to carrying out these functions.

**Table 1.** Characteristics of study participants by province

Characteristics	Ontario *n = 20* *n* (%)	Nova Scotia *n = 21* *n* (%)	British Columbia *n = 15* *n* (%)	Newfoundland & Labrador *n = 12* *n* (%)	*TOTAL* *n = 68* *n* (%)
*Gender* *					
Men	10 (50)	9 (42.9)	4 (36.4)	4 (33.3)	27 (39.7)
Women	10 (50)	12 (57.1)	11 (63.6)	8 (66.7)	41 (60.3)
*Practice Type*					
Fee-for-service	4 (20)	7 (33.3)	6 (40)	5 (41.7)	22 (32.4)
Alternative payment plan**	16 (80)	14 (66.7)	9 (60)	7 (58.3)	46 (67.6)
*Hospital Privileges*					
No	15 (75)	6 (28.6)	3 (20)	5 (41.7)	18 (26.5)
Yes	5 (25)	15 (71.4)	12 (80)	7 (58.3)	49 (73.5)
*Community Size^α^*					
Rural	9 (45)	8 (38.1)	0	3 (25)	20 (29.4)
Small urban	1 (5)	0 (0)	0	0 (0)	1 (1.5)
Urban	8 (40)	13 (61.9)	15 (100)	8 (66.7)	44 (64.7)
Mix	2 (10)	0 (0)	0	1 (8.3)	3 (4.4)
*Years in Practice (mean)*	18.7	15.4	16.9	16.3	16.9

Notes:

*Gender was asked as an open-ended question; **Alternate payment includes all non-fee-for-service or enhanced fee-for-service payment types; *α* – Rural < 10,000 population, Small urban = 10,000–99,999 population, Urban > 100,0000

### Context of taking on pandemic-related leadership roles

Many physicians working in each region and across a variety of practice and remuneration models “stepped-up” to provide leadership based on their existing positions and connections to the broader system or because of their individual expertise:

[The] Physician Lead for our group, he became very involved in the COVID response with our community. My colleague who had the expertise in infection control also got very involved with our local Public Health […]” [ON03].

Many family physicians indicated that they assumed a leadership role because they wanted to respond to the need in their clinic or community: “some of the other people within clinics stepped up simply because they had to […] help coordinate” [ON10]. For some, these roles were formally recognized with official positions on provincial and regional bodies responsible for overseeing the pandemic response: “[…] myself and a few other doctors found ourselves on a task force in the province, to help advise at the primary care health level” [NL03].

For many others in preexisting leadership roles in their clinic or academic-based positions, coordinating the pandemic response became part of their duties:

We all coordinated our response to the pandemic, it was the clinic leads from each of the sites that […] was the core committee making those decisions […]. So, I mean, the response to COVID, of course, was an additional task but it was considered to be a part of that role that I’ve been playing prior to that. [NL08]

Similarly, another physician who held a role as Chief of Family Medicine at the local hospital was called upon to help organize pandemic response programs, such as setting up assessment centres: “So I think because of my Chief role […] I was pulled into a table that planned our local assessment center and that involved a couple of other family physicians, eventually, who are currently working there” [ON12].

Many family physicians who took on more informal roles within their clinic or communities (e.g. members of informal physician or clinical leadership groups) were called upon because of their prior knowledge and experience:

I ended up sort of becoming the de facto COVID lead. You asked if I had a defined title, like, that’s not really a defined title, but I think if you asked anyone at the site, they would say that [I was the COVID lead]. [BC13]

In this example, the physician took on the role of adapting public health directives to operations of the clinic and the needs of the vulnerable patient population it served.

### Leadership functions

This paper outlines three key functions of family physician leadership during the COVID-19 pandemic: conveying knowledge, developing and adapting protocols for primary care practices and advocacy. Each of these functions involve curating and synthesizing information and tailoring communications to specific audiences. These functions require an understanding of the individual needs and the decision-making processes of each audience.

### Conveying knowledge

In all four regions in the study, family physicians were conveyors of knowledge to three key groups. First, they shared information with decision makers in charge of pandemic planning. Family physicians highlighted issues on the frontlines (lack of direct guidance, changing protocols, unmet patient needs, difficulties with coordination and management of patient care, etc.) and in some cases developed and put forward plans for more senior administrators to consider. A family physician in rural Ontario described how a group of physicians developed plans to deal with the distribution of personal protective equipment for administrators to consider and became part of the leadership table as a representative for family physicians:

It was a group of us, about four or five physicians that had come together and thought that, in our opinion, our region was not planning as well as [we] felt they should. More likely in the hospital and integrating with the hospital and community sector. So, there was about four of us that came together and started talking about what we should be doing […]. So, we actually had ourselves positioned on those tables, alongside with our hospital sector as well. So, I would say that a lot of my role, and with some of my close colleagues, was more of a leadership role. [ON05]

In Nova Scotia, a family physician was able to change access to lab testing by coordinating physician concerns and relaying this information to regional managers:

[…] it was one physician who […] had some serious concerns about the lab restrictions […]. So, she wrote me this wonderful email. So, what did I do? I […] sent it up the channels. And by the end of the day, we had a private line, an emergency line for doctors in the community to call in order to get those lab tests done that they needed. [NS07]

Similarly, family physicians provided feedback on plans developed by hospitals and specialists that had implications for their practice and the care of their patients. A family physician described his role in ensuring that family physician concerns were included in plans for obstetrical care:

Should one of our obstetrical patients […] have a COVID infection […] the hospital had a variety of different policies and their own care diagrams and algorithms. So, trying to partake and give input into all of that was another role. [ON13]

Second, family physicians played an important role in educating and supporting other family physicians. A physician in Newfoundland and Labrador described his role in answering questions posed in a physician social media group:

[…] there is a Facebook physician group […] I joined that group actually to partly play a role. So, we were contributing to answering some clinician questions around COVID, as a service [from] our research unit […]. One of [the questions] we worked on was, “how long does the virus survive on surfaces, […] and what sort of […] products can be used to disinfect surfaces?” […] We did a little bit of background work on that and looked to what else has been published around that.” [NL08]

A family physician in Ontario described how she had sifted through the various emails directed toward primary care and identified the important messages to relay to her colleagues:

I had a role in educating my colleagues, or sort of filtering the information because there’s so much coming from so many different organizations […]. It was hard for our colleagues to know what to do. [ON01]

A key element of conveying knowledge to other family physicians was creating the means (e.g. listservs, Facebook groups, Zoom meetings, etc.) to connect with peers. Family physicians also created their own networks specifically to check on well-being and provide peer support to mitigate mental health issues. A family physician in Nova Scotia noted that routine meetings also became an opportunity to support one another:

We now actually spend time and go around and ask each other, how are you doing? We do a mental health and physical health check-in. What's going on? That idea of being very mindful of each other's health. [NS19]

Third, family physicians were a trusted source of information to the public in terms of COVID-19 and restrictions. Family physicians helped distill public health measures within their clinics:

[…] we had a public service announcement […] in our local town paper […]. We met with the town council, we met with the mayor, we met with the council members to explain why we're doing […]. I mean we're the only health centre in this area. So, we endeavoured to ensure that all those players understood why and what we're doing. [NS19]

Similarly, another family physician noted that:

[…] one of the roles […] that many family physicians did play, was to be a source of reputable and informative scientifically-based medical information. So, we did videos for our community television, […] we did videos on how to properly hand wash, we did videos about why COVID is different, why it’s scary, why masks are important. And people found that very, very helpful. We had a lot of positive feedback, people said it was, it was easy to understand, it was coming from somebody they trusted […]. And as the vaccines come, we’re gong to be doing more videos on our community television, about the vaccine. [NS10]

### Developing and adapting protocols for primary care practices

Leadership roles for many family physicians included adapting broad public health directives to the operations of a family practice. Many took the lead in organizing workflows for physicians in their group. A family physician in Ontario noted:

We just honestly took it upon ourselves, we had an emergency meeting, all six physicians, to basically roll out a flow-sheet, like a patient flow care plan on how to manage the incoming calls, to triaging, to who do we bring in? […] if they have infectious symptoms, what do we do? [ON07]

Often, family physicians had to tailor processes to fit the unique needs of their patient population. A family physician in British Columbia described how his clinic needed to re-imagine broad public health guidelines to address the needs of patients at a community-based clinic:

[…] at our addiction clinic, we spent a lot of time […] trying to come up with, okay, well how do we actually operationalize this? How do we take this broad guidance which kind of doesn’t have a lot of detail, and then how can we actually make it applicable and safe for our particular clinic and program? […] We spent hours and hours drafting guidelines and our own protocols […]. [BC12]

### Advocacy

Family physician leadership was also demonstrated through advocacy work. Family physicians have a special understanding of their patient populations, especially individuals with vulnerabilities because of health conditions or personal circumstances. At the health system level, family physicians showed leadership by calling attention to the needs of vulnerable or marginalized communities: “[…] People's social conditions were in many situations quite destabilized in terms of social connections, access to housing, access to food. So, you know […]. to advocate for improved social conditions […]” [NS20]. A family physician in Newfoundland and Labrador recalled working with public health officials and community groups to ensure that public health information was available to people who did not speak English:

[…] we worked closely with the Association for New Canadians [to create] a phone tree to all of their contacts to notify them about what the Public Health guidelines were […]. I was concerned about my population who couldn’t speak English and that they wouldn’t know to wash […] they wouldn’t know to self-isolate on return from their travel. [NL03]

In British Columbia, many physicians described the extra steps they took to provide care for patients with addiction issues. In addition to adapting the delivery of medical care, physicians also addressed broader social care needs and support programs:

I know this sounds silly, but things like renewing driver’s licenses, renewing MSP, like, all that stuff became a problem […]. I think we [providers at the clinic] are often the first people who kind of see those things outside of the people who literally experience them immediately. [BC15]

Family physicians identified specific needs of their patient populations and many provided extra supports. Similar to many family physicians in all regions, a family physician in Ontario identified individuals with mental health needs in his practice who would have a difficult time coping with pandemic-related closures and would likely benefit from proactive outreach:

We’ve had a lot of mental health care patients […] really taking a look at […] which patients really need the support, and having somebody call them and going to see how they are, how they’re doing. [ON08]

### Leadership barriers and supports

Lack of time, lack of remuneration, absence of a clear plan for primary care and inadequate communication with system partners were identified by family physicians as barriers to carrying out leadership functions. In contrast, regional structures that connected individual family physicians to health systems and decision-making bodies supported family physicians in assuming leadership activities.

#### Barriers.

Regardless of whether they were in a formal or informal leadership position, fulfilling these crucial roles consumed a considerable amount of time and often resulted in an added burden on top of an already heavy workload. The description of the workload was similar across regions:

But it was probably like a hundred hours of work in the month of March that I did extra because of COVID […]. Yeah, it was, like I just would get up every day and work from the moment I woke up to when I went to sleep for like, a whole month, basically. [BC02]

Another participant from Nova Scotia said:

[…] we were working almost around the clock for over a month making new protocols and then doing simulations and things to make sure that we were ready and safe, keeping ourselves safe and keeping our patients safe and making all the new protocols”. [NS02]

For physicians paid by alternate payment plans, taking on leadership roles was easier (although this did not mitigate the increase in workload) because it did not reduce time for clinical services (and fee-for-service generated income): “And I sort of took that [the leadership role] on, but I mean, most people wouldn’t do that, it was partly because how I’m paid; I’m paid salary” [BC11]. However, fee-for-service physicians reported not receiving compensation for their leadership duties: “I felt like I was a Public Health […] full-time employee and everyone else at the table [was] being paid except for me” [ON04]. Family physicians noted a need for remuneration for their leadership contributions: “I mean, they need some financial support, for sure. I think you have to recognize how much administrative stuff there is involved in all of this” [ON13].

Leadership activities were hindered by a lack of a clear plan for primary care. As a result, many family physicians exerted considerable additional effort to figure out how their own practices would respond: “We didn’t have any sort of official guidance in those early weeks for sure; it was all pretty grassroots” [BC12]. Individually, without a coordinated plan, physicians organized how they would operate their practices: “[there] was no true guidance really, at that time, from with respect to Public Health in terms of how to manage our family practices” [ON07]. Moreover, the lack of a primary care-specific plan led to tension with local or regional health system administrators who disagreed with the approach proposed by family physicians and contributed to a sense of frustration among family physicians. For example, a participant in British Columbia described how conflicting opinions and previous negative experiences with the Health Authority dampened family physician enthusiasm in responding to subsequent public health directives:

[…] the front-line staff felt that we should be prepared and maybe set a room aside if somebody shows up symptomatic. We felt like they needed to be seen in person and we talked and thought we came up with a plan […]. But then we brought that idea forward to management, they didn’t feel that that was a good idea. And so, yeah, we kind of stopped trying to come up with, respond quickly by coming up with [solutions to] changing Public Health orders. We sort of were told that operational things needed to come from operations and so we would have to sort of, wait to be told and given direction about what to do.” [BC05]

Although family physicians were relied upon to inform their communities about pandemic measures (e.g. governments encouraging patients to ask their family doctor questions about COVID-19 or any other health issues throughout the pandemic), they did not always have the necessary information from health system managers to ensure that messages were consistent. For family physicians who had recognized and assumed the responsibility of communicating to patients or the public, the lack of information support was a source of frustration. A family physician in Nova Scotia who had made a learning resource to inform her community recalled the negative feedback she received from the Health Authority:

[…] the Health Authority said, “Well, it’s kind of okay you said that, but we would have said it a little bit differently.” And it kind of irked me a little bit, I’m like, “Well, then you should have provided me with some kind of media sheet or fact sheet or talking points.” […] If it’s an important role for family doctors to do and, and we are certainly pushed into that, then certainly, some kind of talking points of, “These are things as a Health Authority, we would like family physicians to be saying in your office, and in any media opportunities, so that the same message is being spread across the province.” [NS10]

#### Supports.

Family physician leadership was aided by regional structures that connected individual family physicians to health systems and decision-making bodies. These structures varied across the four cases in the study, depending on local primary care reforms. In Nova Scotia, hospital privileges (which are required of all family physicians in the province) often provided an efficient means to obtain information for many physicians:

“I can appreciate if I was not connected to the family practice office and [emergency department], and I was just a family doc without that connection, it would […] that translation or transmission of information would have been slowed and probably a little bit more fragmented.” [NS19]

In contrast, in British Columbia where hospital admitting privileges are not a requirement, family physicians who did not have formal links to regional health system structures felt they lacked access to relevant information: “As a community clinic we were not and still are not really privy to all of the information that comes through the Health Authority” [BC01]. In Ontario, a family physician who had previously held a formal leadership role at his local hospital noted that his connection to other leaders gave him access to information and helped coordinate primary care activities:

But I used to do the Clinical Lead for primary care […] [and there is a] Chiefs WhatsApp, so like little groups like that where we would say, “Okay, this is what’s going on, what are you doing in your region, this is what is going on in this region.” Like, that kind of spreading of information and communication […]. So, I would say it was my relationship with all the chiefs in each of the hospitals for each of the departments that helped me to facilitate and connect everybody together. [ON05]

Participants noted that regional structures could play an important role in managing the large amount of information directed at family physicians and reduce the burden on individual family physicians. In British Columbia, one of the family physicians discussed the need for the Divisions of Family Practice (community-based groups of family physicians that are funded through a partnership between the Ministry of Health and Doctors of BC to provide needed infrastructure in their regions) to assist with this responsibility:

We talked about how that would be an important service that the Division [of Family Practice] could play […] rather than all of us individually doing that work at every clinic. And that they could be the trusted curator of all of that stuff. [BC01].

The Divisions of Family Practice assumed these responsibilities over time. Pandemic responses that were built upon regional organizational structures provided an efficient and effective means to facilitate family physician leadership through preexisting governance, lines of communication and established relationships. Although Ontario was undergoing a transition from Local Health Integration Networks (LHINs) to Ontario Health Teams (OHTs) when the pandemic began, these organizations served as a useful mechanism for coordination and physician engagement:

So, we had primary care leads in Southwest LHIN that were accountable to each sub-region before. […] as these pandemic tables were being created and launched, said, “Hey, I need these people back, they need to be my conduit for each of these subregions,” right? […] And so, the OHT lingo made it more formal, it created some governance and accountability around what we were already doing, but the relationships for the leadership groups was there […]. It was certainly helpful to have more mature relationships […]. It’s been found to be an effective structure to navigate and communicate with our local leads and having clinicians talk to clinicians seems to be a far more engaging strategy […]. [ON14]

## Discussion

We conducted and analyzed semi-structured qualitative interviews to describe the leadership functions of family physicians in four regions in Canada during the COVID-19 pandemic. Regardless of whether family physicians were appointed to formal roles or acting in an *ad hoc* capacity, the common functions of family physician leadership were to convey knowledge to health system and pandemic response managers, colleagues and patients; to develop and adapt public health measures to primary care practices and specific patient populations; and to advocate for the unique needs of their patient populations. While these functions describe qualities of family physician leadership during the pandemic, they are consistent with the leadership role in the CanMEDS competency framework for family physicians (Tepper and Hawrylyshyn, 2017). As expected with CanMEDS competencies, these leadership functions overlap with medical expert, communicator, collaborator and health advocate roles (Royal College of Physicians and Surgeons of Canada, 2021). They also highlight the core principles of family medicine (College of Family Physicians of Canada, 2021) including the longitudinal relationships between family physicians and patients that allow family physicians to understand the particular needs and challenges of their patient populations and to advocate on their behalf (Bernard *et al.*, 2019; Earnest *et al.*, 2010; Sherin *et al.*, 2019; Soklaridis *et al.*, 2018). As community-based practitioners, family physicians are knowledgeable about local factors and have a unique understanding of the communities they serve and therefore are well-suited to plan and implement community-adaptive procedures for a variety of settings, including shelters, other congregate living settings, outreach programs and family practice clinics. Family physician leadership, along with other core competencies, were key to promoting accessibility of care and patient health during the pandemic.

Our findings have important implications for future pandemic response planning. Many family practices in Canada operate independently as private businesses (Health Canada, 2003), and therefore, pandemic plans need to consider ways of communicating and coordinating with individual practices, especially if family practices do not have existing formal linkages with regional or institutional networks. Pandemic plans need to formally appoint family physicians in leadership positions in regional pandemic response structures and facilitate appointment of leaders in clinics and group practices. Formally appointed, designated leaders convey a sense of authority and accountability (Snell *et al.*, 2016) and are particularly needed in unfamiliar, rapidly unfolding crises, such as a pandemic, that call on physicians to deviate from routine tasks and activities (Paquin *et al.*, 2018). Formal family physician leadership is also needed to enable bidirectional information flow between community-based providers and decision makers. Pandemic plans should include supports that enable family physicians to carry out key roles, including timely access to information, tools to facilitate timely communication to family physicians (e.g. listservs, group chats, etc.), remuneration and coverage for clinical duties. Previous studies of physician leadership have highlighted the need to increase leadership training for physicians (Canadian Medical Association [CMA], 2012; Gallagher *et al.*, 2017; Snell *et al.*, 2016) given the limited coverage of leadership in existing undergraduate medical curricula. While courses and continuing professional development certifications exist for general leadership needs (Canadian Medical Association Joule, 2021; Canadian Society of Physician Leaders, 2021; Jolemore and Soroka, 2017), training targeting the specific skills needed for pandemic leadership should be incorporated into pandemic planning.

In each region, existing models of primary care (i.e. funding and human resource arrangements) influence the degree to which family physicians can participate in leadership and other pandemic response roles. Previous studies support the need to compensate physicians for leadership activities, especially fee-for-service physicians, given their loss of income because of the reduced provision of clinical care (CMA, 2012; Snell *et al.*, 2016). For example, in Nova Scotia, a short-term provincial Income Stability Program was introduced for fee-for-service physicians to ensure that their practices (and professional activities outside clinical care) were supported through loss of income because of canceled procedures or visits (Nova Scotia Medical Services Insurance, 2020). Similarly, the Pandemic Physician Work Disruption Program was introduced in Newfoundland and Labrador to support fee-for-service physicians during periods of service disruption and to help accommodate these physicians to participate in pandemic-related services when needed (e.g. staffing assessment centers, extended clinical hours, emergency room coverage, etc.) (Government of Newfoundland and Labrador, 2021).

This study also highlights how primary care reforms have contributed or detracted from a coordinated pandemic response. Provinces that had implemented reforms that created regional organization structures, such as the OHTs in Ontario (which replace the LHINs) or the Divisions of Family Practice in British Columbia, could take on roles of synthesizing and disseminating evidence, thereby eliminating the need for individual practices to review studies and develop their own workflows. Physicians compensated through alternate payment plans were also better able to adapt to pandemic-related closures. Lastly, regions where physicians were connected to decision-making bodies through network membership or hospital privileges, such as clinics that were under the jurisdiction of a Health Authority, had access to information in a timelier manner and were better able to communicate concerns and advocate for vulnerable groups through a clear line of communication. Communications enabled by hospital privileges also helped coordinate pandemic response between primary and acute care providers.

### Limitations

The four regions across four provinces in Canada included in this study varied in terms of rurality, the number of COVID-19 cases and COVID-19-related deaths, associated impact on hospital and other health system functioning, COVID-19 policy responses, as well as general organization and funding of primary care. However, the variation in these data also provide real-world insight into similarities and differences across systems that are run provincially and have varying pandemic risk exposure. Pandemic experiences and primary care systems in other regions may be different than those in the four regions studied. Despite using maximum variation sampling and a variety of recruitment approaches, some physician perspectives (e.g. solo practitioners) may not be fully captured in our data. We carried out interviews between October 2020 and June 2021. Our data may not fully capture the leadership roles of family physicians during the vaccination phase, as access to vaccinations were available only for priority populations during the data collection phase and did not become available to the general adult population until June 2021 and to children between five and 12 years in December 2021. Given the rapid changes through the various stages of the pandemic, the interview data may be subject to recall bias (Coughlin, 1990). Similarly, data may have been influenced by social desirability bias, that is, physicians may have felt obligated to give responses that are expected of them or portrayed them favorably (Bergen and Labonté, 2020).

## Conclusions

Family physicians “stepped up” and played important leadership roles in the COVID-19 pandemic response. Family physician leadership functions included conveying knowledge to decision makers, their peers and their patient populations and communities; adopting public health measures to primary care settings; and advocating for vulnerable peoples. These leadership functions carried out by family physicians build upon their established long-term relationships with patients and their understanding of the health and social needs of their patient populations. Pandemic plans for primary care should include formal family physician leadership appointments as well as supports, such as training, communication aids, coordination and compensation. Engaging family physicians through leadership roles is integral to ensuring that primary care practices can adapt to changing public health guidelines during the various stages of a pandemic, ensuring uninterrupted and safe access to care for patients.

## Appendix. Interview guide

In this study, we want to gain a better understanding of the roles of family physicians during a pandemic. By roles, we mean specific tasks and/or responsibilities that family physicians are asked or required to do during the stages of the pandemic.

First, I would like to ask some general background questions:

- How long have you been practicing as a family physician?
- In which communities do you currently practice and how would you describe them in terms of urban or rural?
- What is your practice model? How are you paid?
- Can you tell me about the nature of your current practice in terms of where you work, for example, do you provide care in a community-based practice? ED? Long term care home? Hospital as a hospitalist? Home visits?
- Do you belong to organized networks or physician groups? Which ones?
- Do you have privileges at any hospital or other facility?
- Do you have any contractual or other obligations to any health care organizations (e.g. local hospital, medical school, long term care home or other facility)?
- What is your gender?
- Do you routinely care for dependent family members?

In the next set of questions, I would like to focus on the period from January to mid-March 2020. During this time, we first started to hear about COVID-19 and cases were starting to show up in Canada:

1. During the PRE-CLOSURE period, could you describe what ACTUAL roles or functions you carried out for patients who had (or were suspected to have) COVID-19 as well as your other patients?
  - [Probe based on responses to first set of questions]: Community based practice, LTC, ED, hospital, other.
2. Can you tell me about what supports were available to you to help carry out these roles? What barriers did you experience?
  - Probe: Access to PPE, funding, communications with networks/groups/hospital.
3. Looking at the list of PROPOSED roles during the pre-closure stage, what supports would be needed to enable you to carry out these roles? What barriers did you experience?
  - Probe: Access to PPE, funding, communications with networks/groups/hospital.
4. Are there other roles you think family physicians could or should have played during the PRE-CLOSURE stage? What supports would be needed to carry out those roles?
  - Probe: roles at different facilities
  - Probe: Access to PPE, funding, communications with networks/groups/hospital
5. Is there anything else about this stage that you would like to share with us?

Now, let us consider the CLOSURE Stage, from mid-March to mid-May. During this time, schools and most businesses were closed and physicians were advised to close practices except for essential visits:

1. During the CLOSURE period, could you describe what ACTUAL roles or functions you carried out for patients who had (or were suspected to have) COVID-19 as well as your other patients?
  - Probe: Access to PPE, funding, communications with networks/groups/hospital
2. Can you tell me about what supports were available to you to help carry out these roles? What barriers did you experience?
  - Probe: Access to PPE, funding, communications with networks/groups/hospital
3. Looking at the list of PROPOSED roles during the CLOSURE stage, what supports would be needed to enable you to carry out these roles? What barriers did you experience?
  - Probe: Access to PPE, funding, communications with networks/groups/hospital
4. Are there other roles you think that family physicians could or should have played during the CLOSURE stage? What supports would be needed to carry out those roles?
  - Probe: roles at different facilities
  - Probe: Access to PPE, funding, communications with networks/groups/hospital
5. Is there anything else about this stage that you would like to share with us?

Now, I would like to consider the PHASED RE-OPENING Stage, from mid-May to today. During this time, many businesses re-opened in some form. School opening plans were introduced and physicians were advised to limit in-person care to essential visits:

1. During the PHASED RE-OPENING period, could you describe what ACTUAL roles or functions you carried out for patients who had (or were suspected to have) COVID-19 as well as your other patients? What barriers did you experience?
  - Probe: Access to PPE, funding, communications with networks/groups/hospital
2. Looking at the list of PROPOSED roles during the PHASED RE-OPENING stage, what supports would be needed to enable you to carry out these roles? What barriers did you experience?
  - Probe: Access to PPE, funding, communications with networks/groups/hospital
3. What other roles do you feel family physicians could or should have played during the PHASED RE-OPENING stage? What supports would be needed to carry out those roles?
  - Probe: roles at different facilities
  - Probe: Access to PPE, funding, communications with networks/groups/hospital
4. Is there anything else about this stage that you would like to share with us?
5. To date we have been able to avoid a scenario where the emergency departments and hospitals are overwhelmed by COVID-19 cases. If this were to happen, what additional roles should family physicians have?
  - Probe: roles at different facilities
6. What supports would be needed to allow family physicians to fulfill these roles? What barriers exist to these roles?
  - Probe: Access to PPE, funding, communications with networks/groups/hospital

For the final set of questions, I would like to switch gears a bit:

- Can you tell me about other non-physician responsibilities you have in your life?
- How do your non-physician responsibilities influence the roles that you are able to play in a pandemic? What supports are needed to allow family physicians with other responsibilities to fulfill pandemic roles? What barriers did you experience?
- Thinking of your gender […]. Does your gender influence the roles that you are able to play in a pandemic? What supports are needed to allow all genders to fulfill these roles? What barriers did you experience?
- Should we go through additional pandemic stages and family physician roles evolve, may we contact you about doing another interview in the future about those additional stages and roles? You can decide whether you want to participate at that time.
- Those are all the questions I have. Is there anything you would like to add?
